# 非小细胞肺癌驱动基因突变及靶向治疗的研究进展

**DOI:** 10.3779/j.issn.1009-3419.2014.10.07

**Published:** 2014-10-20

**Affiliations:** 1 063009 唐山，河北联合大学研究生院 Department of Postgraduate, Hebei United University, Tangshan 063009, China; 2 063000 唐山，河北联合大学附属医院 Department of Respiratory Medicine, the Affiliated Hospital of Hebei United University, Tangshan 063000, China

**Keywords:** 肺肿瘤, 驱动基因, 靶向治疗, Lung neoplasms, Driver gene, Targeted therapy

## Abstract

肺癌是癌症死亡的重要原因。驱动基因的发现使肿瘤治疗不再“一刀切”。靶向治疗改变了癌症药物治疗的现状成为“带眼睛的子弹”，其疗效可见并为肺癌治疗带来一场革命。驱动基因及靶向治疗已经成为非小细胞肺癌（non-small cell lung cancer, NSCLC）新的代名词。2013年中国美国临床肿瘤学会（American Society of Clinical Oncology, ASCO）年会发布了关于NSCLC的11种驱动基因突变频率，本文将就此11种NSCLC驱动基因突变的结构、功能及靶向药物治疗进行阐述。

## Abstract (en)

Lung cancer is the leading cause of cancer-related mortality in the worldwide. The discovery of drive gene makes tumor treatment is no longer "one-size-fits-all". Targeted therapy to change the present situation of cancer drugs become "bullet" with eyes, the effect is visible and bring a revolution in the treatment of lung cancer. The diver gene and targeted therapy have became the new cedule of non-small cell lung cancer (NSCLC). Society of Clinical Oncology (ASCO) has showed 11 kinds of diver genes. Here, we review the functional and structural characteristics and the targeted therapy in the 11 kinds of driver gene mutations.

肺癌是癌症死亡的重要原因，发病率高，在我国肺癌发病率呈逐年上升趋势，且年均增长1.63%^[1]^。美国癌症协会（American Cancer Society）对2014年美国癌症死亡人数、发病率、死亡率和存活率进行预测评估显示：新增肺癌患者占全部肿瘤患者的27%（男）和29%（女）；死亡肺癌患者占全部肿瘤患者的28%（男）和26%（女）^[2]^。

肺癌的靶向治疗已经成为世界趋势并且提高了反应率（response rate, RR）、延长了总体生存时间（overall survival, OS）和无进展生存时间（progression free survival, PFS），但是靶向药物使用一段时间后机体会产生原发性或获得性耐药，其中一个重要原因就是驱动基因发生突变。2013年中国美国临床肿瘤学会（American Society of Clinical Oncology, ASCO）年会上非小细胞肺癌（non-small cell lung cancer, NSCLC）驱动基因突变频率最新数据显示：表皮生长因子受体（epidermal growth factor receptor, EGFR）为10%-35%；鼠类肉瘤病毒癌基因（Kirsten rate sarcoma viral oncogene homolog, KRAS）为15%-25%；间变型淋巴瘤激酶（anaplastic lymphoma kinase, ALK）为3%-7%；v-akt鼠胸腺瘤病毒原癌基因1（v-akt murine thymoma viral oncogene homolog 1, AKT1）为1%；v-raf鼠类肉瘤病毒癌基因B1（v-raf murine sarcoma viral oncogene homolog B1, BRAF）为1%-3%；原癌基因人类表皮生长因子受体（human epidermal growth factor receptor-2, HER2）为2%-4%：丝裂原活化蛋白激酶1（mitogen-activated protein kinase kinase 1, MEK1）为1%；v-ras成神经细胞瘤病毒癌基因（v-ras neuroblastoma viral oncogene homolog, NRAS）为1%；磷脂酰肌醇-3-激酶催化亚单位基因（phosphatidylinositol-3-kinase catalytic subunit gene, PIK3CA）为1%-3%；原癌基因转染重排（rearranged during transfection, RET）为1%-2%；c-ros原癌基因1酪氨酸激酶（c-ros oncogene 1 receptor tyrosine kinase, ROS1）为1%-2%。因而有必要研究驱动基因突变，为靶向药物治疗的发展奠定基础。

## 1 上游靶点突变

### 1.1 *EGFR*突变

EGFR为跨膜的酪氨酸激酶受体，为ErbB家族（HER1/ErbB1、HER2/ErbB2、HER3/ErbB3、HER4/ErbB4）成员之一。表皮生长因子（epidermal growth factor, EGF）、转化生长因子α（transforming growth factor-α, TGF-α）、角化细胞内分泌因子（amphiregulin, AREG）等配体与EGFR结合^[3]^，酪氨酸激酶磷酸化激活信号传导通路RAS/RAF/MEK/MAPK、PI3K/AKT/mTOR、STATs，参与细胞增殖、分化、血管形成、转移，其机制主要为：①逃逸细胞凋亡；②新生血管（肿瘤缺氧微环境）；③抵制抑制生长信号；④侵袭和转移[上皮间质转化（epithelial mesenchymal transition, EMT）]；⑤生长信号自我供给；⑥基因组不稳定。⑦无限复制（端粒酶的持续表达）。因此EGFR酪氨酸激酶抑制剂（EGFR tyrosine kinase inhibitor, EGFR-TKI）如吉非替尼作用的靶点已成为肿瘤药物治疗的热门靶点。

*EGFR*突变在此11种驱动基因中突变频率最高，常见于女性、非吸烟、肺腺癌、亚洲患者。并且EGFR在大部分肿瘤中过度表达：NSCLC≥50%、头颈部鳞癌≥90%、食管癌30%-70%^[4]^，其高表达与不良预后和放疗抵抗密切相关。*EGFR*突变基因位于外显子18-21，突变包括细胞外的缺失突变和细胞内TK区的体细胞突变。缺失突变主要见于747-750位框架氨基酸，体细胞点突变最常见于L858R^[5]^，其他突变包括△LRE、G719R、二次突变T790M等。机体获得性耐药第一代EGFR抑制剂主要见于：①T790M、T854A、D761Y、L747S突变；②MET传导通路改变；③ EMT；④其他：*PTEN*（抑癌基因）丢失和胰岛素样生长因子受体（insulin-like growth factor receptor, IGFR）激活等^[6]^。第二代EGFR抑制剂为阿法替尼，与第一代相比作用范围更广。LUX-lung3和LUX-lung3临床试验数据表明：第二代抑制剂可用于突变患者，也可作用于对第一代抑制剂（吉非替尼）不敏感的患者。此试验有利于指导临床靶向治疗，给对第一代抑制剂耐药及不敏感的肺癌患者带来又一次机会。2013年中国的胸部肿瘤研究组CTONG公布0806结果，数据表明*EGFR*野生型患者不可使用EGFR抑制剂。该结果为EGFR抑制剂排除了适应症，有利于指导临床靶向用药。

### 1.2 *HER2*突变

HER2属于酪氨酸激酶感受器（receptor tyrosine kinase, RTKs）家族，编码调节细胞增殖的跨膜受体。RTKs包含EGFR/ERBB1、HER2/ERBB2/NEU、HER3/ERBB3、和HER4/ERBB4。

HER2位于17号染色体，在一些肿瘤中发现其拷贝数扩增^[7]^。HER2受体不存在与其相匹配的配体，而与其他受体组成同源二聚体或异源二聚体，优先接受EGFR和HER3。HER2可激活RAS/RAF/MEK（细胞增殖）传导通路和PI3K/AKT/mTOR（细胞存活）^[8]^。HER2在NSCLC中突变频率为2%-4%，主要见于不吸烟、女性、腺癌患者；最常见的突变位于外显子20的插入突变，增强了HER2激酶活性和信号转导，导致细胞增殖、侵袭和肿瘤发生，此插入突变不能与HER2扩增同时发生。

针对HER2的靶向药物为曲妥珠单抗，对存在*HER2*突变的肺癌患者有效。一项回顾性分析存在外显子20 HER2插入突变的65例NSCLC患者的临床病理、治疗方案、患者结局的结果^[9]^表明：曲妥珠单抗的控制率为93%，无进展生存时间为5.1个月，早期患者的平均生存时间为89.6个月，晚期患者为22.9个月。此研究证明了在肺腺癌中*HER2*基因突变筛选的重要性和HER2靶向药物的治疗潜力。

## 2 RAS/RAF/MEK/MAPK传导通路

### 2.1 *KRAS*突变

RAS蛋白是下游生长因子受体信号的中心调节位点，调节细胞增殖、分化。*RAS*基因家族包括*KRAS*、*HRAS*、*NRAS*，此三种基因高度同源但功能大相径庭。*RAS*基因突变与多种肿瘤发病机制相关，在缺乏生长因子信号的条件下也可激活RAS GTP酶，导致细胞持续增殖。15%-25%的NSCLC存在*KRAS*突变，大于90%的突变位于外显子2（密码子12或13）。与EGFR不同，*KRAS*突变常见于吸烟、腺癌患者^[10]^，20%见于白种人，5%见于黄种人。*KRAS*突变被认为与第一代TKIs和常规化疗耐药相关。一项Ⅲ期临床试验TRIBVTE研究将晚期NSCLC患者随机分为试验组和对照组，试验组为常规化疗+埃罗替尼，对照组为常规化疗+安慰剂，将其做为一线治疗^[11]^。试验结果表明*KRAS*突变能减少试验组患者的肿瘤进展期和OS（OS HR=2.1; 95%CI: 1.1-3.8）。然而一项KRAS突变Ⅲ期试验回顾性分析（BR.21、SATURN以及FLEX）表明：患者受益于埃罗替尼和西妥昔单抗的大小取决于患者的*KRAS*野生型突变^[12, 13]^。Planck等^[14]^研究表明：*EGFR*突变及*EGFR*/*KRAS*野生型存在不同的基因组改变、不同临床病理特征、不同总生存期。可见通过对肿瘤患者进行准确的基因检测，分析临床病理特征，找到最适人群，可使治疗效果最大化。

### 2.2 *NRAS*突变

*NRAS*的突变频率为1%，主要见于吸烟的肺腺癌患者^[15]^，主要突变形式为G12C、G12R、G12S、G12A、G12D、Q61K、Q61L、Q61R、Q61H。NRAS突变导致NRAS信号通路被激活。目前尚未研发出针对NRAS的靶向药物，但临床前期试验^[15]^表明*NRAS*突变对MEK抑制剂敏感。Haarberg等研究^[16]^显示：HSP90抑制剂XL888对*NRAS*突变的黑色素瘤敏感，可以抑制体外培养细胞生长，使细胞停留于G2期-M期。但对于*NRAS*突变的NSCLC患者的研究还是冰山一角，其针对*NRAS*突变的靶向药物需要进一步研究。

### 2.3 *BRAF*突变

*BRAF*基因位于染色体7q34编码丝/苏氨酸蛋白激酶。做为RAS/有丝分裂原激活蛋白激酶信号通路的成员，BRAF为KRAS的下游传导通路，直接磷酸化MEK，最后引起ERK磷酸化，使细胞增殖和存活。*BRAF*突变频率为1%-3%，主要见于重度吸烟的肺腺癌患者^[17]^。目前*BRAF*突变类型有V600E、G469A、D594G等40多种形式，在NSCLC患者中V600E突变最常见。与*KRAS*突变相似，*BRAF*突变在结直肠癌的突变频率（8%-20%）明显高于NSCLC。

BRAF的靶向药物为BRAF抑制剂，目前已研发出多种BRAF抑制剂，但对NSCLC疗效差。第一代BRAF抑制剂代表为索拉菲尼，为第一个已有临床试验证明的RAF激酶抑制剂。第二代以PLX4032为代表，具有高度选择性，并在V600E突变的黑色素瘤Ⅲ期临床试验中取得明显疗效。2013年5月食品药品监督管理局（Food and Drug Administration, FDA）批准Tafinlar（dabrafenib）（BRAF抑制剂）治疗肿瘤表达*BRAF* V600E基因突变黑色素瘤患者，但对肺癌患者效果不明显，研究者正在寻找针对BRAF肺癌治疗的靶向药物。

### 2.4 *MEK1*突变

MEK1又名MAP2K1，是丝/苏氨酸蛋白激酶，调节MAP激酶信号通路，参与MAP信号传导并参与多个细胞过程如细胞增殖、细胞分化、转录调节。MEK1突变频率为1%，主要见于肺腺癌，最常见的突变为C121S、Q56P、K57N、D67N。SEQ ID NO:2^[18]^表明C121S突变产生野生型的MEK1蛋白，并对RAF和MEK抑制剂耐药。Q56P突变发生在MEK1激酶区外，此突变可增强MEK1激酶活性，并可与野生型EGFR和ALK同时存在。K57N和D67N突变同样在MEK1激酶区外，导致MAPK信号通路开放，但K57N对小分子的非ATP竞争抑制剂（AZD6244）敏感^[19]^。*MEK1*突变促进了第二代MEK抑制剂的研究，第二代MEK抑制剂可用于*MEK1*突变的患者，疗效值得期待。

## 3 PI3K/AKT/mTOR传导通路

### 3.1 *PIK3CA*突变

PI3K-AKT-mTOR通路首次发现于1990s，为EGFR下游靶点，与细胞存活和抗凋亡相关。PI3K是脂质激酶家族成员之一，参与细胞生长、增殖、分化、运动，其结构为异源二聚体。PI3K包含2个亚基，1个为85 kDa的调节亚基，另1个为110 kDa的催化亚基。*PIK3CA*基因编码催化亚基p110α。PI3K使细胞内膜的PI(4, 5)P2（磷脂酰肌醇4, 5-二磷酸）转变为PI(3, 4, 5)P3[磷脂酰肌醇（3, 4, 5）-三磷酸肌醇]。PI(3, 4, 5)P3招募下游信号蛋白如AKT到细胞膜导致其他蛋白活性增强。*PIK3CA*突变频率为1%-3%，突变的肺鳞癌患者多于肺腺癌患者，与吸烟史无关，突变多位于外显子9和外显子12，常见于E542K、E545K、E545Q、H1047L、H1047R。*PIK3CA*突变与*EGFR*突变可同时出现，并且可在获得性耐药EGFR TKI患者中出现。Bendell等^[20]^认为PI3K抑制剂可应用于体内实体肿瘤如早期直结肠癌、乳腺癌、肺癌，表现一定的抗癌活性。PI3K抑制剂（GDC-0941、XL-147、PX-866、BKM 120）已在体外证明有效，并在临床试验证明中^[21]^。

### 3.2 *AKT1*突变

AKT是PI3K下游的关键信号，可激活肿瘤发生；AKT又名蛋白激酶B，是调节PI3K信号传导的丝-苏氨酸激酶，由AKT1、AKT2、AKT3组成^[22]^。*AKT1*突变编码的蛋白酶B已在乳腺癌、结肠癌、卵巢癌中发现。*AKT1*突变率为1%，主要见于鳞癌患者，其突变频率最高的为Glu17Lys，此突变发生于*AKT1*基因的PH结构域并改变磷酸肌醇结合袋，使蛋白激酶B具有独立于PI3K的活性。

AKT1的靶向药物为AKT抑制剂。ATP竞争AKT抑制剂已被报道有更高的脱靶效应，因此变构的AKT抑制剂已被研发，目的在于研发出特异性抑制剂。例如AKT变构抑制剂结合AKT区的PH结构域，阻止AKT定位到细胞膜并随后被激活。MK-2206为AKT变构抑制剂，磷酸化AKT的308位苏氨酸和473位丝氨酸残基，在体内外均有抗肿瘤活性，从而促进了临床试验的进展。

## 4 融合基因

### 4.1 *ALK*融合

棘皮类微管相关样蛋白-4（echinoderm microtubuleassociated protein-like 4, *EML4*）的基因在内含子13断裂与位于内含子19的*ALK*基因偶联形成*EML4-ALK*融合基因，此融合基因共包含3, 926个碱基对，编码1, 059个氨基酸^[23]^。*EML4-ALK*融合异常激活下游的信号传导通路如MAPK、PI3K、信号转换器、STAT导致细胞增殖、侵袭，抑制细胞凋亡^[21]^。*ALK*融合常见于年轻、男性、非吸烟或少量吸烟的腺癌患者，临床特点为转移早，常见于心包转移、胸膜转移、胸腔内外淋巴结转移、肝转移等。针对该靶点的药物为克唑替尼，属于3-苄氧-2氨基吡啶类抑制剂，与ALK酶的ATP结合位点相结合。克唑替尼已经获得FDA批准可用于ALK^+^的NSCLC患者，此次批准的根据来源于I期临床试验^[24]^的数据，该试验表明：使用克唑替尼的总有效率为57%，PFS为6个月的概率为72%。但是一部分ALK^+^患者存在原发性耐药，一部分使用克唑替尼9个月的ALK^+^患者也可发展为继发性耐药。Doebele等^[25]^研究11例ALK^+^的NSCLC患者克唑替尼耐药的分子机制，研究表明：4例（36%）患者在ALK的酪氨酸激酶区发生二次突变，其中2例患者出现G1269A；1例患者存在耐药突变表现为ALK融合拷贝数增加（copy number gain, CNG）；1例患者存在*EGFR*突变，ALK融合消失；2例患者显示*KRAS*突变，其中1例患者不存在*ALK*融合；1例患者*ALK*融合消失但未发现其他可识别驱动基因改变；2例患者ALK^+^耐药机制不明。可知ALK^+^的NSCLC患者克唑替尼耐药的分子机制复杂，需加大样本量进一步研究。

30%使用克唑替尼的患者早期出现脑转移，原因在于克唑替尼不能通过血脑屏障，其在脑脊液中的药物浓度是外周血的0.002, 6倍。目前针对ALK的第二代TKI处于研究临床试验阶段，第二代TKI对*ALK*野生型突变和对克唑替尼耐药的二次突变有更强的亲和力，能改善药物代谢并能进入血脑屏障，而且对于其他的酪氨酸激酶的靶点如ROS、Met同样有抑制作用。针对ALK的第二代TKI代表之一为LDK378，2013年3月美国FDA认定LDK378是对于ALK^+^ NSCLC的进展期和对克唑替尼耐药的患者是突破性疗法，目前正处于Ⅲ期临床试验，预期在2014年出台管理文件^[26]^。

### 4.2 *RET*融合突变

*RET*原癌基因于1985年因激活NIH-3T3细胞DNA重排而首次发现，位于10q11.2外显子12，在细胞内、外及跨膜区编码酪氨酸激酶。*RET*融合突变在NSCLC患者中突变频率为1%-2%。RET在正常条件下表达很低，但在*RET*融合条件下表达明显增高，且*RET*突变与NSCLC其它驱动基因突变相排斥不能同时发生。Wang等^[27]^研究具有*RET*融合突变的NSCLC患者的临床特点，结果显示：*RET*融合突变常见于年轻人（≤60岁）、不吸烟、早期淋巴结转移、低分化的实体亚型。目前尚没有针对RET靶点的RET抑制剂用于*RET*融合突变的NSCLC临床试验，但抗RET的酪氨酸激酶抑制剂（靶点不是*RET*融合基因）已经在NSCLC患者中进行评估。目前正处于试验阶段的vandetanib、sunitinib及sorafenib并不是特殊的RET抑制剂，而是多重激酶抑制剂^[28]^，其试验结果值得期待。

### 4.3 *ROS1*融合突变

ROS1为酪氨酸激酶，属于胰岛素受体家族，位于6q16-6q22，激活ROS1可开通信号传导通路STAT3、PI3K/AKT、RAS/MAPK/MEK。ROS1在NSCLC患者中突变频率为1%-2%，其临床特征与ALK易位相似，主要见于不吸烟或轻度吸烟（< 10包/年）、年轻的肺腺癌患者。*ROS1*融合突变不可与其他驱动基因突变同时出现。ROS1与ALK高度同源，在ATP结合区存在77%的相同序列。使用ALK激酶抑制剂在细胞系和组织中可检测到ROS1融合蛋白，ALK抑制剂TAE664对细胞系HCC78敏感，此细胞系在使用TAE664刺激后可存在*SLC34A2-ROS1*融合基因，并可在BaF3细胞中表达FIG-ROS融合蛋白。2012年欧洲临床肿瘤协会年会（European Society for Medical Oncology, ESMO）和美国临床肿瘤学会（American Society of Clinical Oncology, ASCO）会议显示对ROS1重排的NSCLC使用克唑替尼2个月后的反应率为57%，疾病控制率为80%^[29]^。目前AP26113（NCT01449461）和ASP3026（NCT01284192）两个临床试验正在进行中，主要目的为评估*ROS1*融合突变的患者使用第二代ALK抑制剂的安全性及药物效果，其试验结果值得期待。

## 5 总结

近年来*EGFR*及其他驱动基因的发现给肿瘤治疗带来一场改革，使肿瘤治疗不再“一刀切”，通过对患者驱动基因准确检测可实行特异性和规范化治疗。基因检测的普及可使肿瘤治疗真正进入个体化时代。

驱动基因突变与NSCLC患者的病理类型、年龄、性别、TNM分期、远处转移、吸烟史等相关。国际上关于驱动基因突变研究的研究对象大部分为白种人及黑种人，黄种人所占比例偏少，因此在国内进行驱动基因突变的研究是十分有意义的。患者在使用靶向药物治疗过程中发生原发性或获得性耐药，根据不同耐药机制解决耐药问题，将成为我们日后的关注点。

对NSCLC驱动基因进行深入研究可为靶向治疗提供理论依据，有望阐明NSCLC发生的分子机制并找到靶向治疗的最适人群，使治疗效果最大化。驱动基因突变在NSCLC中的意义越来越受到学者的关注，靶向药物治疗已成为趋势。尽管许多基因突变已被发现且靶向治疗存在种种优势，但肿瘤的靶向治疗还存在很多不足，需要我们进一步的研究。
